# Prognostic and therapeutic implications of a low aortic valve calcium score in patients with low-gradient aortic stenosis

**DOI:** 10.1093/ehjci/jeae276

**Published:** 2024-10-29

**Authors:** D Juhász, M Vecsey-Nagy, Á L Jermendy, B Szilveszter, J Simon, B Vattay, M Boussoussou, D Dávid, P Maurovich-Horvát, B Merkely, A Apor, L Molnár, E Dósa, M Rakovics, J Johnson, A Manouras, A I Nagy

**Affiliations:** Heart and Vascular Centre, Semmelweis University, 68. Varosmajor u, 1122-Budapest, Hungary; Heart and Vascular Centre, Semmelweis University, 68. Varosmajor u, 1122-Budapest, Hungary; Department of Radiology and Radiological Science, Medical University of South Carolina, Charleston, SC, USA; Heart and Vascular Centre, Semmelweis University, 68. Varosmajor u, 1122-Budapest, Hungary; Heart and Vascular Centre, Semmelweis University, 68. Varosmajor u, 1122-Budapest, Hungary; Department of Radiology and Radiological Science, Medical University of South Carolina, Charleston, SC, USA; Heart and Vascular Centre, Semmelweis University, 68. Varosmajor u, 1122-Budapest, Hungary; Heart and Vascular Centre, Semmelweis University, 68. Varosmajor u, 1122-Budapest, Hungary; Heart and Vascular Centre, Semmelweis University, 68. Varosmajor u, 1122-Budapest, Hungary; Medical Imaging Centre, Semmelweis University, Budapest, Hungary; Heart and Vascular Centre, Semmelweis University, 68. Varosmajor u, 1122-Budapest, Hungary; Heart and Vascular Centre, Semmelweis University, 68. Varosmajor u, 1122-Budapest, Hungary; Heart and Vascular Centre, Semmelweis University, 68. Varosmajor u, 1122-Budapest, Hungary; Heart and Vascular Centre, Semmelweis University, 68. Varosmajor u, 1122-Budapest, Hungary; Centre for Translational Medicine, Semmelweis University, Budapest, Hungary; Department of Statistics, ELTE Eötvös Loránd University, Faculty of Social Sciences, Budapest, Hungary; Department of Medicine Huddinge, Karolinska Institutet, Stockholm, Sweden; Department of Medicine Huddinge, Karolinska Institutet, Stockholm, Sweden; Heart and Vascular Centre, Semmelweis University, 68. Varosmajor u, 1122-Budapest, Hungary

**Keywords:** low-gradient aortic stenosis, TAVI (transcatheter aortic valve implantation) aortic valve calcium score

## Abstract

**Aims:**

Low-gradient (LG) aortic stenosis (AS) poses a diagnostic challenge. Aortic valve calcium score (AVCS) assessment has emerged as a complementary diagnostic method when echocardiography provides discordant results. However, the diagnostic and prognostic values of AVCS in LGAS have not been thoroughly studied. Our aims in this study were to investigate the prognostic importance of AVCS in LGAS and to assess whether symptomatic patients with LGAS and low AVCS may benefit from aortic valve intervention (AVI).

**Methods and results:**

A total of 327 symptomatic patients (78.5 ± 7.3 years, 51% women) with severe AS defined by the aortic valve area who underwent computed tomography for transcatheter aortic valve intervention (TAVI) planning were enrolled. AVCS was measured. AVCS < 2000AU in men and < 1200 AU in women was considered a low AVCS. A total of 243 patients had high gradient (HG) and 84 had LGAS. A low AVCS was present in 25 (10%) patients with HG and 34 (40%) with LGAS. Over a median follow-up period of 4.9 years, 194 deaths occurred. In multivariate analysis, AVCS was a significant independent predictor of all-cause mortality among patients with HGAS [adjusted hazard ratio (aHR): 2.317; CI: 1.104–4.861; *P* = 0.026] but not among those with LGAS (aHR: 0.848; CI: 0.434–1.658; *P* = 0.630). After propensity score matching between patients who underwent AVI and those who were medically treated, AVI (94% TAVI) was a significant and independent predictor of survival among LGAS patients with a low AVCS even after adjustment for clinical variables (aHR: 0.102, CI: 0.028–0.369; *P* < 0.001).

**Conclusion:**

The prevalence of a low AVCS is much higher in patients with LGAS than in those with HGAS. In patients with symptomatic severe LGAS, a low AVCS does not entail a better prognosis. AVI is equally beneficial in LGAS patients with a high or low AVCS, similarly to those with HGAS.

## Graphical Abstract

**Graphical Abstract:**
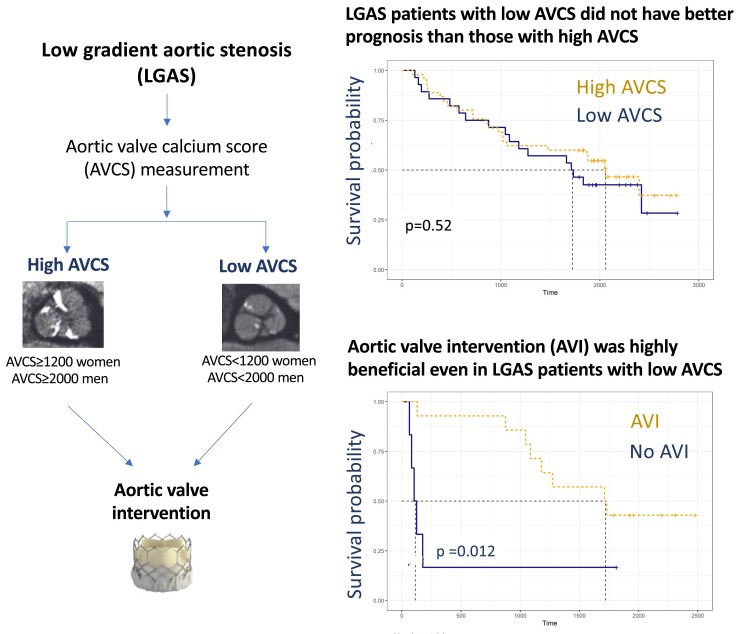
AVCS has a limited value in the risk stratification of LGAS. Patients with a low AVCS equally benefit form AVI, like those with a high AVCS. AVCS, aortic valve calcium score; AVI, aortic valve intervention; LGAS, low-gradient aortic stenosis.

## Introduction

The burden of aortic valve calcification shows strong correlation with the haemodynamic parameters of aortic stenosis (AS).^1–3^ Accordingly, aortic valve calcium score (AVCS) by computed tomography (CT) has emerged as a diagnostic tool to complement echocardiography in patients in whom Doppler measurements provide conflicting data or there is clinical uncertainty about the severity of AS.^4^ In these situations, AVCS may help the grading of severity and thereby guide further clinical management. Diagnostic cut-off values for severe AS were previously established, and incorporated into the diagnostic pathways, based on which aortic valve replacement (AVR) is currently recommended.^4^ However, recent data demonstrated that patients with low-gradient aortic stenosis (LGAS) (i.e. a transvalvular mean pressure gradient (aortic mean (transvalvular) gradient, AMG) < 40 mmHg despite a calculated aortic valve area (AVA) ≤ 1 cm^2^) tended to have a lower aortic valve calcium load than those with high gradients (HGs).^5,6^ The prevalence of an AVCS below the diagnostic threshold among patients with echocardiographically severe LGAS can be as high as 50%.^5^

The pathomechanism behind senile AS is a combination of progressive fibrosis and calcification leading to valve thickening and reduced valve motion. Although calcium deposition is a hallmark of severe AS, the degree of mineralization vs. fibrosis appears to be influenced by several factors, including age, sex, valve phenotype, and other underlying diseases like cardiac amyloidosis (CA).^7,8^ Thus, certain patient cases are characterized by a predominantly fibrotic phenotype with a relatively less degree of calcium deposition, despite severely narrowed aortic valve orifice. In these patient cases, AVCS might underestimate the severity of AS and therefore delay appropriate management.

In addition to its diagnostic role, AVCS also has an established predictive value for patient outcome. However, recent reports suggested that the prognostic value of AVCS was weaker among patients with LGAS when compared with those with HG severe or moderate AS.^5^

We set out to investigate to prognostic value of AVCS among patients with LGAS and to assess whether symptomatic patients with LGAS and an AVCS below the threshold may benefit from AVR, in particular transcatheter aortic valve implantation (TAVI).

## Methods

### Study design

**Figure 1:**
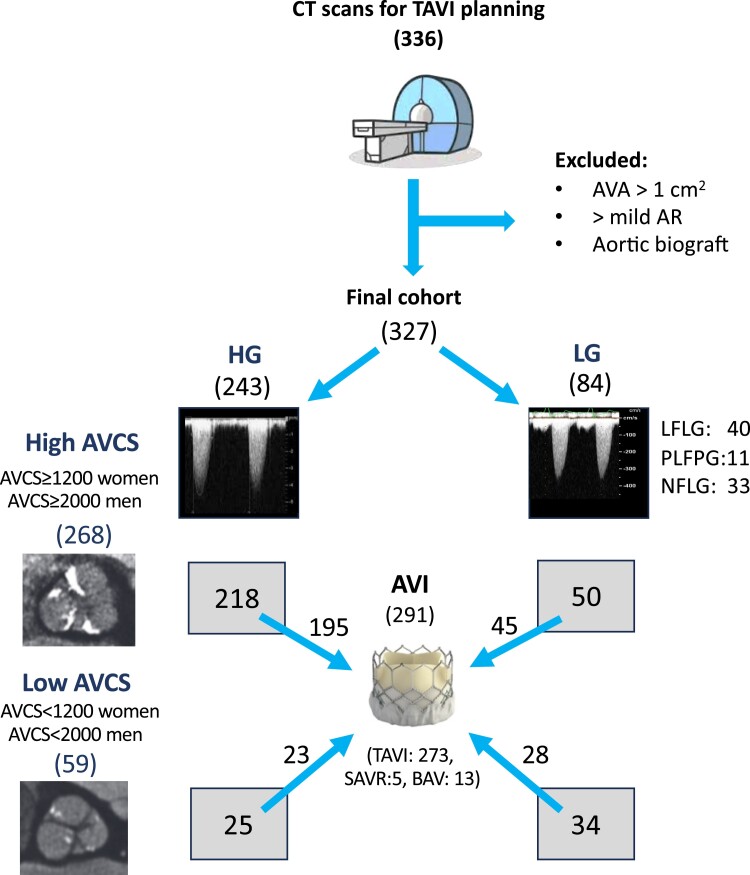
Study population. CT, computed tomography; TAVI, transcatheter aortic valve implantation; AVA, aortic valve area by Doppler echocardiography; AR, aortic regurgitation; HG, high gradient; LG, low gradient; AVCS, aortic valve calcium score; AVI, aortic valve intervention; SAVR, surgical aortic valve replacement; BAV, balloon aortic valvuloplasty; LFLG, classical low-flow low-gradient AS; PLFLG, paradoxical low-flow low-gradient AS; NFLG, normal-flow low-gradient AS.

This was a retrospective single-centre observational study. Consecutive patients with severe AS based on echocardiography who underwent cardiac CT angiography for TAVI planning at the Semmelweis University Heart and Vascular Centre between 22 February 2016 and 8 April 2019 were retrospectively enrolled. Patients with more than mild aortic regurgitation, biograft in the aortic position, or a non-severe AS by echocardiography were excluded (*Figure 1*). Demographic and clinical data were collected from electronic medical records. Coronary artery disease (CAD) was defined as previous myocardial infarction or coronary revascularization in the patient records. The study protocol complied with the Declaration of Helsinki and was approved by the Institutional Review Board of Semmelweis University Heart and Vascular Centre. The need for informed consent was waived because of the retrospective nature of the study.

### Echocardiography

All patients underwent comprehensive Doppler echocardiography prior to CT, performed by one of four echocardiographers with over 10 years of experience in echocardiography, ensuring homogeneous examination quality. Echocardiographic measurements were performed in accordance with the current recommendations.^9^ The AMG was calculated by using the modified Bernoulli formula and stroke volume (SV) was measured by the Doppler method of the left ventricle (LV) outflow tract and indexed (SVi) to body surface area (BSA). The AVA was calculated using the continuity equation as absolute AVA or AVA indexed (AVAi) to BSA. Severe AS was defined as AVA ≤ 1 cm^2^ or AVAi ≤ 0.6 cm^2^/m^2^.

### Cardiac CT acquisition

We prospectively performed ECG-triggered CT using a 256-slice CT scanner (Philips Healthcare, Best, The Netherlands, 270 ms rotation time, tube voltage of 100–120 kV). Native images were acquired with 2 mm slice thickness (no overlap) using iterative reconstruction (iDose4 or IMR, Philips Healthcare, Cleveland, OH, USA). AVCS measurements were performed offline on a dedicated workstation equipped with commercially available software (HeartBeat CS, Philips Healthcare, Cleveland, OH, USA) by the Agatston method.^10^ A single investigator conducted all measurements, identifying both valve leaflet and annular calcifications on axial slices. Female patients with an AVCS < 1200 AU and male patients with an AVCS < 2000AU were considered as having a low AVCS.^4^

For the current investigation, we also analysed the contrast-enhanced CT images to visualize the aortic valve in multiple planes and to confidently exclude calcifications in non-valvular anatomic regions such as the ventricular outflow tract, mitral valve annulus, and coronary arteries. The contrast-enhanced images were acquired retrospectively using ECG-gated helical CT angiography of the aorta (from the level of the thoracic inlet to the level of the femoral head) and the heart during a single breath-hold, in the cranio-caudal direction using the same scanner.

### Patient subgroups

On echocardiographic examination, patients with an AMG ≥ 40 mmHg were categorized as high-gradient (HG) AS and those with an AMG < 40 mmHg as LGAS. Within the LGAS cohort, patients were further classified into classical low-flow low-gradient (LFLG), paradoxical LFLG (PLFLG), or normal-flow low-gradient (NFLG) AS based on the LV ejection fraction (EF) and the SVi (i.e. LFLG AS: EF < 50%, SVi ≤35 mL/m^2^; PLFLG AS: EF ≥ 50%, SVi ≤ 35 mL/m^2^; NFLG AS: SVi > 35 mL/m^2^, irrespective of the LVEF).

According to the CT results, female patients with an AVCS < 1200 AU and male patients with an AVCS < 2000AU were considered as having a low AVCS (*Figure 1*).

### Study end-points

The primary study end-point was all-cause mortality. To assess the prognostic value of AVCS, the overall survival of patients with high and low AVCSs was compared. This analysis was also performed confined to patients who underwent aortic valve intervention (AVI). To assess the prognostic value of AVCS during the natural history of AS (i.e. when it has an impact on decision-making about AVI), survival under medical treatment was analysed. For this analysis, patients who underwent AVI were censored at the time of AVI (but it was not an end-point) and those who did not undergo AVI were followed up until death (which was an end-point). This analysis is provided in the Supplementary data online, *File*. To assess the prognostic benefit of interventional vs. medical treatment, AVI was used as a time-dependent covariate in the Cox regression models.

Follow-up data, including information about AVR or balloon aortic valvuloplasty (BAV), as well as survival data, were collected from the national health care administration system ensuring that no patient was lost to follow-up.

### Statistical analysis

Continuous variables were tested for normality using the Shapiro–Wilk test. Normally distributed continuous variables are presented as mean ± SD, whereas non-normally distributed variables are presented as median (interquartile ranges). Categorical variables are presented as frequencies and percentages. Variables were compared between groups using Welch-corrected F-tests, except for survival after CT where the Kruskal–Wallis test was used. In the latter case, raw survival times are presented; a comparison of survival under medical treatment produced essentially identical results. Determinants of a low AVCS were assessed using a multiple logistic regression model.

Kaplan–Meier curves were created to illustrate differences in survival probability between groups. Survival analyses were also performed to assess the importance of AVCS, and the role of AVI, using multivariate Cox proportional hazards analyses, adjusted for clinically relevant variables. The variables used for the adjustment were age, sex, the presence of hypertension, diabetes, atrial fibrillation, CAD, estimated glomerular filtration rate (eGFR), and LVEF. AVI was used as a time-dependent variable. The parameters included in the multivariate models were predefined based on clinical relevance.

For the assessment of the effect of AVI on outcome, to limit the baseline differences between patients who did and those who did not undergo intervention, propensity score matching was used (1:3 in the LG group and 1:6 in the HG group). The parameters used to calculate the propensity score included age, sex, LVEF, eGFR, and history of atrial fibrillation and diabetes mellitus. The success of the propensity score match was assessed by comparing the distribution of patient characteristics and propensity scores in the matched samples.

In order to identify potential alternative AVCS cut-off values in the LGAS cohort that can predict a good prognosis, we compared the estimated median survival of patients with low and high AVCSs at various AVCS cut-offs using Cox-regression models. For men, the tested cut-off values were always set 800 AU higher than for women. To assess whether there was any AVCS cut-off, under which no benefit of AVI can be demonstrated, the size of the effect of AVI on survival was tested at various AVCS thresholds, separately in the LG and HG cohorts.

## Results

### Patient characteristics

The composition of the study population is summarized in *Figure 1*. The final study cohort comprised of 327 patients, the mean age was 78.5 ± 7.3 years, 51% were female, and 107 patients (33%) had reduced LVEF (<50%). Sixteen patients had a bicuspid aortic valve, and no patient had rheumatic disease. A total of 243 patients had high-gradient AS (AVA ≤ 1 cm^2^ or AVAi ≤ 0.6 cm^2^/m^2^ and AMG ≥ 40 mmHg) and 84 had LGAS (AVA ≤ 1 cm^2^ or AVAi ≤ 0.6 cm^2^/m^2^ and AMG < 40 mmHg). In the latter group, 40 patients had classical LFLG AS (LVEF < 50%, SVi ≤ 35 mL/m^2^), 11 had PLFGL AS (LVEF ≥ 50%, SVi ≤ 35 mL/m^2^), and 33 patients had NFLG AS (SVi > 35 mL/m^2^; independent of LVEF). The absolute AVA was above 1 cm^2^ in 7 patients, and these patients were classified as severe AS based on AVAi ≤ 0.6 cm^2^/m^2^. All patents were symptomatic. Baseline demographic and clinical data of the study population are provided in *Table 1*, and further characterization according to the LVEF and the type of LGAS is provided in Supplementary data online, *Table S1*.

**Table 1:** Baseline demographic, clinical, and imaging data of the patient cohort stratified by the aortic mean transvalvular gradient and aortic valve calcium score

	HGAS (*n* = 243)			LGAS (*n* = 84)		
	High AVCS (*n* = 218)	Low AVCS (*n* = 25)	*P*-value	High AVCS (*n* = 50)	Low AVCS (*n* = 34)	*P*-value
Demographic data and comorbidities						
Age (years)	78.7 (± 7.3)	80.6 (± 6.1)	0.1697	76.7 (± 8.3)	78.0 (± 6.2)	0.4056
Female (*n*, %)	120 (55.0)	13 (52.0)	0.7787	22 (44.0)	11 (32.4)	0.2843
Hypertension (*n*, %)	190 (88.4)	21 (87.5)	0.9049	46 (92.0)	31 (91.2)	0.896
Diabetes mellitus (*n*, %)	86 (40.0)	10 (41.7)	0.8786	25 (50.0)	21 (61.8)	0.2915
Atrial fibrillation (*n*, %)	81 (37.7)	10 (41.7)	0.7144	26 (52.0)	15 (44.1)	0.4842
CAD (*n*, %)	72 (34.8)	14 (60.9)	**0**.**0243**	15 (30.6)	20 (58.8)	**0**.**0114**
eGFR (mL/min/1.73 m^2^)	60.4 (± 19.9)	60.6 (± 19.2)	0.9543	61.4 (± 22.0)	48.5 (± 19.1)	**0**.**0057**
LV EF <50% (*n*, %)	47 (21.6)	3 (12.0)	0.1931	33 (66.0)	24 (70.6)	0.6612
Echocardiography data						
AMG (mmHg)	55.1 (± 14.3)	45.9 (± 8.3)	**<0**.**001**	33.0 (± 5.9)	27.6 (± 7.2)	**<0**.**001**
AVA (cm^2^)	0.67 (± 0.19)	0.75 (± 0.18)	0.0532	0.77 (± 0.19)	0.82 (± 0.18)	0.2187
LV EF (%)	55.3 (± 11.0)	59.6 (± 10.2)	**<0**.**001**	42.1 (± 13.9)	43.4 (± 13.8)	**<0**.**001**
Bicuspid aortic valve	10 (4.6%)	0 (0%)	0.276	5 (10.0%)	1 (2.9%)	0.222
CT data						
AVCS men (AU)	4003 (2884–5117)	1458 (1352–1620)	**<0**.**001**	3058 (2529–4282)	1499 (1355–1732)	**<0**.**001**
AVCS women (AU)	2434 (1824–3586)	800 (568–971)	**<0**.**001**	2012 (1342–2654)	977 (784–1081)	**<0**.**001**

Continuous data are presented as mean ± standard deviation or median and interquartile ranges, whereas categorical variables are shown as numbers and percentages. *P* values <0.05 are highlighted in bold. HGAS: AMG ≥ 40 mmHg and AVA ≤ 1 cm^2^ or AVAi ≤ 0.6 cm^2^/m^2^; LGAS: AMG < 40 mmHg, AVA ≤ 1 cm^2^ or AVAi ≤ 0.6 cm^2^/m^2^; high AVCS: AVCS ≥ 1200 AU for women and ≥2000AU for men; low AVCS: AVCS < 1200 AU for women and <2000AU for men.

AS, aortic stenosis; HGAS, high-gradient AS; LGAS, low-gradient AS; AVCS, aortic valve calcium score; CAD, coronary artery disease; eGFR, estimated glomerular filtration rate; AMG, aortic mean gradient; AVA, aortic valve area; LV EF, left ventricular ejection fraction; CT, computed tomography; AU, Agatston unit.

### AVCS in the study population and its determinants

A below cut-off AVCS was present in 25 patients (10%) with HGAS and in 34 patients (40%) with LGAS. Within the LGAS group, the rates of prevalence of a low AVCS among patients with classical LFLG, PLFLG, and NFLG AS were 50%, 36%, and 30%, respectively (*P* = 0.223).

The median AVCS value was lower among patients with LGAS than in those with HGAS (median 1826; IQR: 1338, 2872 vs. median 2774; IQR: 1916, 4185; *P* < 0.001). Within the LGAS cohort, patients with PLFLG (median AVCS: 1505; IQR: 1234, 2597) had the lowest median AVCS, followed by the classical LFLG (median AVCS: 1778; IQR: 1339, 2621) and NFLG (median AVCS: 2277; IQR: 1351, 2912) subgroups (overall *P* < 0.001; LFLG vs. PLFLG *P* < 0.001; LFLG vs. NFLG *P* < 0.001; PLFLG vs. NFLG *P* < 0.001).

Patients with a low AVCS had a lower LVEF, were more likely to have CAD, and had a lower AMG, despite similar AVA. Interestingly, in the LGAS subgroup, patients with a low AVCS had worse renal function than those with a high AVCS. The prevalence of a low AVCS tended to be lower among patients with bicuspid valves, although the difference was not statistically significant, and the median AVCS values of the bicuspid and tricuspid valves were similar (3479 and 2528, respectively; *P* = 0.104) (*Table 1* and Supplementary data online, *Table S1*).

Multiple regression analysis, including age, sex, LVEF, eGFR, comorbidities, and echocardiographic indices of AS severity, identified the presence of CAD [odds ratio (OR): 2.881, CI: 1.375–6.034, *P* = 0.005], the LVEF (OR: 1.038, CI: 1.006–1.070, *P* = 0.020), and the AMG (OR: 0.888, CI: 0.852–0.927, *P* < 0.001) as independent predictors of a below cut-off AVCS (see Supplementary data online, *Table S2*). Of note, AVA did not entail a predictive value (*P* = 0.769).

### Aortic valve interventions

All patients were recommended TAVI and interventions were actually performed in 291 patients. A total of 273 patients (94%) underwent TAVI, and 5 patients who were not suitable for TAVI underwent surgical AVR. BAV alone was performed in 13 patients (either as a bridge to TAVI or as palliative treatment because the expected lifespan of the patient was less than one year owing to comorbidities), and one patient underwent BAV and subsequent TAVI. Neither AVR nor BAV was performed in 36 patients, either because the patients refused the procedure or because they died on the waiting list for TAVI (*Figure 1*).

Of the 291 interventions, 240 were performed in patients with a high AVCS and 51 in patients with a low AVCS; of the latter, 28 interventions were performed in patients with a low AVCS and LGAS. Thus, 90% of patients with a high AVCS, 86% of patients with a low AVCS, and 82% of those with a low AVCS LGAS underwent AVI. As provided in *Table 2*, a comparison of the patients receiving different therapies, in terms of demographic characteristics, comorbidity profile, and imaging data, including the prevalence of a low AVCS, revealed no significant differences between the groups. The survival of patients who underwent AVR was better than that of those who had BAV or received medical therapy (*P* < 0.001).

**Table 2:** Demographic characteristics, comorbidities, and imaging data of the population stratified by treatment

	AVR (*n* = 278)	BAV only (*n* = 13)	No intervention (*n* = 36)	*P*-value
Demographic data and comorbidities				
Age (years)	78.3 (± 7.1)	79.2 (± 11.1)	79.4 (± 7.3)	0.851
Female (*n*, %)	141 (50.9)	5 (35.7)	20 (55.6)	0.216
BMI (kg/m^2^)	28.1 (± 5.1)	27.4 (± 6.4)	28.2 (± 6.1)	0.647
Hypertension (*n*, %)	245 (88.8)	14 (100.0)	29 (87.9)	0.239
Diabetes mellitus (*n*, %)	124 (44.9)	3 (21.4)	15 (45.5)	0.147
Atrial fibrillation (*n*, %)	113 (40.9)	6 (42.9)	13 (39.4)	0.823
CAD (*n*, %)	100 (37.6)	7 (50.0)	14 (42.4)	0.675
eGFR (mL/min/1.73 m^2^)	59.9 (± 20.0)	56.4 (± 17.0)	56.1 (± 24.2)	0.879
LV EF <50% (*n*, %)	89 (32.1)	6 (42.9)	12 (33.3)	0.562
Echocardiography data				
AMG (mmHg)	48.5 (± 16.3)	50.9 (± 14.7)	44.6 (± 15.8)	0.188
AVA (cm^2^)	0.71 (± 0.19)	0.71 (± 0.29)	0.70 (± 0.21)	0.910
AVAi (cm^2^/m^2^)	0.37 (± 0.10)	0.39 (± 0.13)	0.39 (± 0.13)	0.980
LV EF (%)	52.5 (± 13.0)	52.3 (± 13.9)	51.5 (± 13.9)	0.824
CT data				
Low AVCS (*n*, %)	48 (17.3)	3 (21.4)	8 (22.2)	0.880
Survival after CT (days)	1863 (988–2199)	502 (263–871)	285 (99–1060)	**<0**.**001**

Continuous data are presented as mean ± standard deviation or median and interquartile ranges, whereas categorical variables are shown as numbers and percentages. Variables were compared between groups using Welch-corrected F-tests, except for survival after CT, where the Kruskal–Wallis test was used. In the latter case, raw survival times are presented; a comparison of survival under medical treatment yielded essentially identical results. *P* values <0.05 are highlighted in bold.

AVR, aortic valve replacement; BAV, balloon aortic valvuloplasty; BMI, body mass index; CAD, coronary artery disease; eGFR, estimated glomerular filtration rate; LV EF, left ventricular ejection fraction; AMG, aortic median gradient; AVA, aortic valve area; AVAi, AVA indexed to body surface area; CT, computed tomography; AVCS, aortic valve calcium score.

### Patient outcome

During a median follow-up period of 4.9 [2.0;5.8] years, 194 (59%) patients died. A total of 162 deaths occurred in the high AVCS (60%) cohort, and 32 deaths were reported in the low AVCS (54%) cohort; of the latter deaths, 22 deaths occurred in the low AVCS LGAS (65%) group.

### The prognostic value of AVCS in patients with HG and LGAS

**Figure 2:**
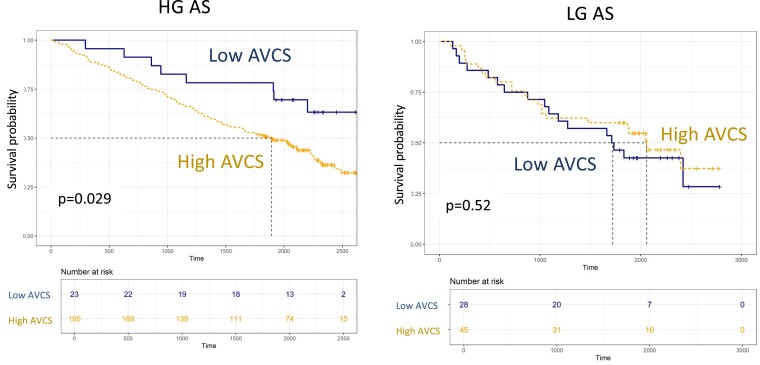
The prognostic value of AVCS in HG and LG AS. Survival probability in patients who underwent AVI, stratified by low and high AVCSs. A) survival among patients with HGAS; B) survival among patients with LGAS. AVCS, aortic valve calcium score; AVI, aortic valve intervention; HGAS, high-gradient AS; LGAS, low-gradient AS.

To assess the prognostic value of AVCS on survival, a COX proportional hazard analysis was performed. This analysis was performed in the entire cohort, and also including only those patients who underwent intervention (*Table 3* and *Figure 2*). The two analyses provided essentially similar results. In HGAS, a high AVCS was a significant independent predictor of worse survival (HR: 1.939; CI: 1.019–3.690; *P* = 0.044, for the entire cohort; HR: 2.177; CI: 1.062–4.462; *P* = 0.034 when only interventionally treated patients were included) even after adjustment for demographic and clinical variables, including age, sex, LVEF, comorbidities (hypertension, diabetes, atrial fibrillation, and CAD), and GFR [adjusted hazard ratio (aHR): 1.979, CI: 1.019–3.843; *P* < 0.044 for the entire cohort; aHR: 2.317; CI: 1.104–4.861; *P* = 0.026, when only interventionally treated patients were included]. However, among patients with LGAS, AVCS did not entail a significant prognostic ability either in univariate (entire cohort—HR: 0.733, CI: 0.417–1.287; *P* = 0.279; medically treated patients excluded—HR: 0.816; CI: 0.436–1.529; *P* = 0.526) or in multivariate analysis (entire cohort—aHR: 0.762, CI: 0.413–1.404; *P* = 0.383; medically treated patients excluded—aHR: 0.848; CI: 0.434–1.658; *P* = 0.630). An analysis of alternative cut-off values that can differentiate prognosis in the LGAS cohort failed to identify any AVCS cut-off values with a prognostic value (see Supplementary data online, *Table S3*). With regard to the prognostic value of AVCS for survival under medical treatment, the results were essentially similar. AVCS was a significant predictor of survival in the HG cohort but not in the LG cohort (see Supplementary data online, *Table S4*), and we could not identify any cut-off value in the LG cohort that could predict a better prognosis.

**Table 3:** Predictive value of aortic valve calcium score for survival among patients with high-gradient aortic stenosis and low-gradient aortic stenosis (interventionally treated patients only)

	Univariate		Multivariate	
Variables	HR (CI)	*P*-value	HR (CI)	*P*-value
High-gradient aortic stenosis				
Age (years)	1.018 (0.991–1.046)	0.188	1.018 (0.990–1.047)	0.209
Female sex	0.893 (0.626–1.274)	0.532	0.909 (0.621–1.330)	0.624
LV EF (%)	0.996 (0.981–1.012)	0.650	1.010 (0.992–1.028)	0.269
Hypertension	0.915 (0.533–1.572)	0.748	0.660 (0.360–1.209)	0.178
Diabetes mellitus	1.483 (1.039–2.117)	0.030	1.511 (1.027–2.225)	0.036
Atrial fibrillation	1.167 (0.811–1.679)	0.406	1.095 (0.751–1.595)	0.638
CAD	1.142 (0.792–1.647)	0.477	1.254 (0.852–1.844)	0.251
eGFR (mL/min/1.73 m^2^)	0.985 (0.976–0.994)	0.001	0.983 (0.973–0.993)	0.001
High AVCS	2.177 (1.062–4.462)	0.034	2.317 (1.104–4.861)	0.026
Low-gradient aortic stenosis				
Age (years)	1.023 (0.976–1.072)	0.343	0.997 (0.941–1.055)	0.910
Female sex	0.913 (0.479–1.743)	0.783	1.034 (0.502–2.129)	0.927
LV EF (%)	1.004 (0.981–1.026)	0.761	0.996 (0.971–1.022)	0.749
Hypertension	1.211 (0.373–3.932)	0.750	1.383 (0.388–4.921)	0.617
Diabetes mellitus	0.864 (0.462–1.615)	0.646	0.809 (0.392–1.669)	0.566
Atrial fibrillation	2.885 (1.500–5.548)	0.001	2.842 (1.438–5.616)	0.003
CAD	1.040 (0.550–1.965)	0.905	1.162 (0.551–2.450)	0.693
eGFR (mL/min/1.73 m^2^)	0.981 (0.966–0.996)	0.015	0.984 (0.966–1.001)	0.068
High AVCS	0.816 (0.436–1.529)	0.526	0.848 (0.434–1.658)	0.630

HGAS: AMG ≥40 mmHg and AVA ≤1 cm^2^ or AVAi ≤0.6 cm^2^/m^2^; LGAS: AMG <40 mmHg, AVA ≤1 cm^2^ or AVAi ≤0.6 cm^2^/m^2^.

AS, aortic stenosis; HGAS, high-gradient AS; AMG, mean transvalvular gradient; AVA, aortic valve area; AVAi, aortic valve area indexed to body surface area; LGAS, low-gradient AS; AU, Agatston unit; HR, hazard ratio; CI, confidence interval; LV EF, left ventricular ejection fraction; CAD, coronary artery disease; eGFR, estimated glomerular filtration rate; AVCS, aortic valve calcium score.

Separate analyses of the predictive value of AVCS among patients with various types of LGAS (LFLG, PLFLG, and NFLG) are provided in the Supplementary data online, *File* (see Supplementary data online, *Figure S1*).

### Propensity score–matched comparison of AVI vs. medical management in patients with LGAS and a low AVCS

**Figure 3:**
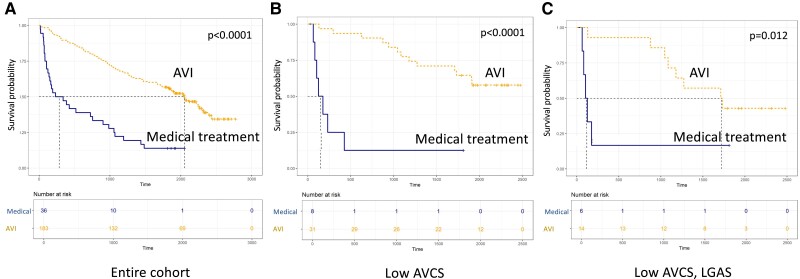
Survival probability of patients with AVI or those under medical treatment. A) in the entire propensity-matched cohort; B) among patients with a low AVCS; C) among patients with a low AVCS and LGAS. AVCS, aortic valve calcium score; AVI, aortic valve intervention; HGAS, high-gradient AS; LGAS, low-gradient AS.

After propensity score matching of patients who underwent AVI and those who received only medical therapy, 219 patients (183 AVI and 36 medical) remained for comparison. Demographic data of the matched cohorts are provided in Supplementary data online, *Table S5*. Kaplan–Meier survival curves according to the type of treatment showed that irrespective of the aortic valve calcification load, patients who underwent AVI had better survival than those who were treated medically (*Figure 3*).

*Table 4* shows the predictors of mortality in uni- and multivariate analyses in the entire matched cohort, among patients with a low AVCS and patients with a low AVCS and LGAS. Time-dependent AVI was a significant independent predictor of survival in all three groups (entire cohort—HR: 0.202, CI: 0.143–0.286; *P* < 0.001; low AVCS—HR: 0.138, CI: 0.044–0.436; *P* = 0.001; low AVCS and LGAS—HR: 0.301, CI: 0.098–0.925; *P* = 0.036) even after adjustment for demographic and clinical variables, including age, sex, LVEF, comorbidities (hypertension, diabetes, atrial fibrillation, and CAD), and GFR (entire cohort—aHR: 0.178, CI: 0.118–0.269; *P* < 0.001; low AVCS—aHR: 0.072, CI: 0.022–0.236; *P* < 0.001; low AVCS and LGAS—aHR: 0.102, CI: 0.028–0.369; *P* < 0.001). Further predictors of adverse outcome included the presence of diabetes, atrial fibrillation, and lower GFR.

**Table 4:** The effect of aortic valve intervention as a time-dependent variable on survival

	Univariate		Multivariate	
Variables	HR (CI)	*P*-value	HR (CI)	*P*-value
In the entire propensity-matched cohort				
Age (years)	1.021 (0.996–1.047)	0.100	1.009 (0.981–1.039)	0.522
Female sex	0.979 (0.716–1.337)	0.892	0.926 (0.645–1.329)	0.676
LV EF (%)	1.006 (0.994–1.017)	0.338	1.013 (0.999–1.028)	0.063
Hypertension	0.837 (0.534–1.311)	0.437	0.640 (0.387–1.057)	0.081
Diabetes mellitus	1.460 (1.069–1.995)	**0**.**018**	1.600 (1.084–2.360)	**0**.**018**
Atrial fibrillation	1.396 (1.017–1.915)	**0**.**039**	1.403 (1.002–1.964)	**0**.**049**
CAD	1.090 (0.794–1.496)	0.596	0.988 (0.675–1.446)	0.950
eGFR (mL/min/1.73 m^2^)	0.987 (0.979–0.994)	**<0**.**001**	0.984 (0.975–0.993)	**<0**.**001**
AVI (time-dependent)	0.202 (0.143–0.286)	**<0**.**001**	0.178 (0.118–0.269)	**<0**.**001**
Among patients with low aortic valve calcium score (propensity-matched cohort)				
Age (years)	1.005 (0.932–1.083)	0.903	1.049 (0.949–1.160)	0.351
Female sex	0.956 (0.412–2.219)	0.916	0.794 (0.267–2.357)	0.678
LV EF (%)	0.990 (0.963–1.019)	0.503	1.003 (0.960–1.049)	0.885
Hypertension	0.621 (0.217–1.773)	0.373	1.236 (0.239–6.397)	0.801
Diabetes mellitus	1.177 (0.509–2.721)	0.703	2.558 (0.832–7.866)	0.101
Atrial fibrillation	2.492 (1.050–5.912)	**0**.**038**	3.829 (1.244–11.785)	**0**.**019**
CAD	0.481 (0.213–1.087)	0.078	0.338 (0.113–1.011)	0.052
eGFR (mL/min/1.73 m^2^)	0.971 (0.952–0.990)	**0**.**003**	0.988 (0.967–1.010)	0.281
AVI (time-dependent)	0.138 (0.044–0.436)	**0**.**001**	0.072 (0.022–0.236)	**<0**.**001**
Among patients with low aortic valve calcium score and low-gradient aortic stenosis (propensity-matched cohort)				
Age (years)	0.989 (0.891–1.098)	0.837	0.932 (0.798–1.088)	0.522
Female sex	0.957 (0.379–2.420)	0.927	0.255 (0.036–1.790)	0.676
LV EF (%)	1.024 (1.003–1.045)	**0**.**025**	1.030 (0.981–1.081)	0.063
Hypertension	0.728 (0.444–1.195)	0.209	2.152 (0.293–15.795)	0.081
Diabetes mellitus	0.673 (0.267–1.694)	0.400	1.357 (0.344–5.354)	**0**.**018**
Atrial fibrillation	2.396 (0.979–5.865)	0.056	1.561 (0.271–9.002)	**0**.**049**
CAD	0.380 (0.158–0.915)	**0**.**031**	0.291 (0.079–1.068)	0.950
eGFR (mL/min/1.73 m^2^)	0.967 (0.944–0.991)	**0**.**007**	0.959 (0.919–1.002)	**<0**.**001**
AVI (time-dependent)	0.301 (0.098–0.925)	**0**.**036**	0.102 (0.028–0.369)	**<0**.**001**

HGAS: AMG ≥ 40 mmHg and AVA ≤ 1 cm^2^ or AVAi ≤ 0.6 cm^2^/m^2^; LGAS: AMG < 40 mmHg, AVA ≤ 1 cm^2^ or AVAi ≤ 0.6 cm^2^/m^2^; high AVCS: AVCS ≥ 1200 AU for women, and ≥ 2000AU for men; low AVCS: AVCS < 1200 AU for women and <2000AU for men. *P* values <0.05 are highlighted in bold.

AVI, aortic valve intervention; AVCS, aortic valve calcium score; AS, aortic stenosis; HGAS, high-gradient AS; AMG, mean transvalvular gradient; AVA, aortic valve area; AVAi, aortic valve area indexed to body surface area; LGAS, low-gradient AS; AU, Agatston unit; HR, hazard ratio; CI, confidence interval; LV EF, left ventricular ejection fraction; CAD, coronary artery disease; eGFR, estimated glomerular filtration rate.

A comparison of the size of the effect of AVI among different subsets of patients based on the AVCS and AMG revealed no significant differences in the magnitude of AVI effect on survival, and AVI was equally beneficial across all patient profiles (*Table 5*).

**Table 5:** The effect of aortic valve intervention as a time-dependent variable on survival depending on the aortic valve calcium score and mean transvalvular gradient in the propensity-matched cohort

	Unadjusted		Adjusted	
	HR (CI)	*P*-value	HR (CI)	*P*-value
Entire cohort	0.202 (0.143–0.143)	**<0**.**001**	0.178 (0.118–0.118)	**<0**.**001**
High AVCS	0.138 (0.044–0.044)	**0**.**001**	0.072 (0.022–0.022)	**<0**.**001**
Low AVCS	0.218 (0.152–0.152)	**<0**.**001**	0.200 (0.130–0.130)	**<0**.**001**
LGAS low AVCS	0.301 (0.098–0.098)	**0**.**036**	0.102 (0.028–0.028)	**<0**.**001**
LGAS high AVCS	0.105 (0.031–0.031)	**<0**.**001**	0.018 (0.004–0.004)	**<0**.**001**
HGAS low AVCS	NA	NA	NA	NA
HGAS high AVCS	0.226 (0.154–0.154)	**<0**.**001**	0.202 (0.130–0.130)	**<0**.**001**

*P* values <0.05 are highlighted in bold. AVCS, aortic valve calcium score; AMG, aortic median gradient; HR, hazard ratio; CI, confidence interval; LGAS, low-gradient aortic stenosis; HG, high-gradient aortic stenosis.

**Figure 4:**
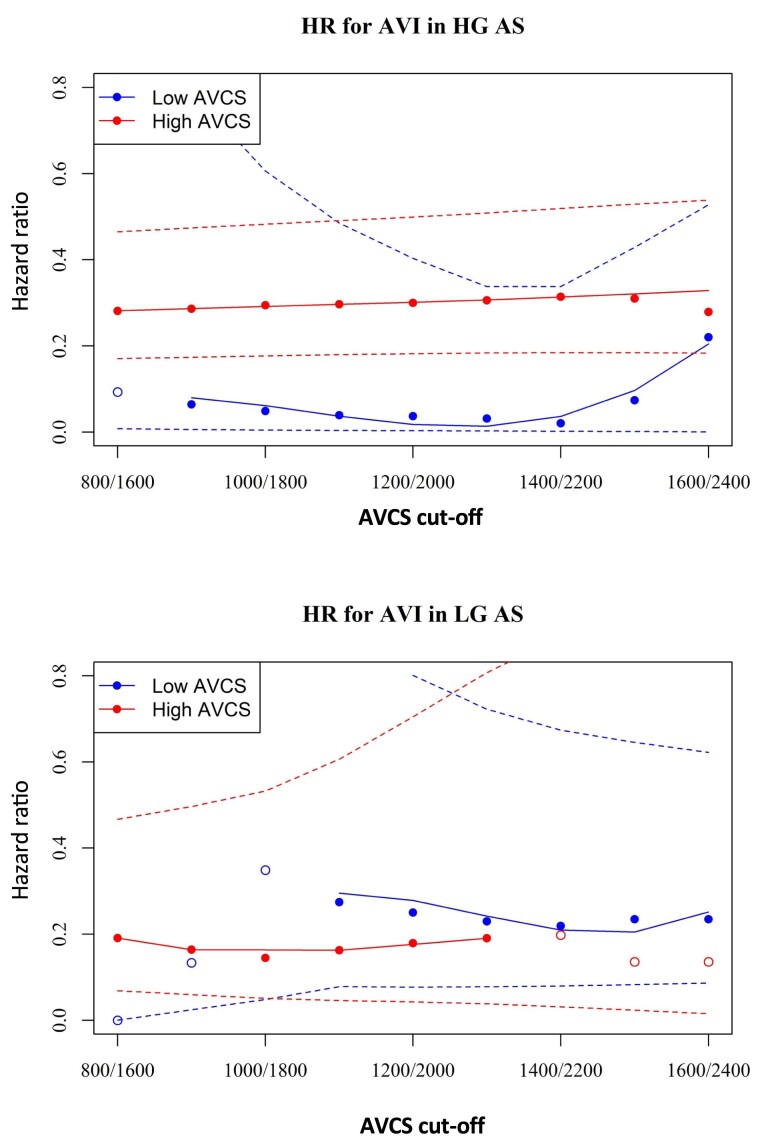
The effect size of AVI to reduce mortality among patients with low and high AVCSs, dependent on the cut-off values used to define ‘low’ and ‘high’ AVCSs. The HR of AVI was tested at various AVCS cut-off pairs for women and men. The filled circles indicate a significant effect of AVI, and the empty circles indicate a non-significant effect of AVI. AVI remained beneficial (HR < 1) across the spectrum of AVCS cut-offs, and the size of the effect was independent of the actual AVCS values. At cut-off values set at the lower and higher margins of the AVCS range, where the case numbers were more limited, no statistically significant benefit of AVI could be demonstrated. AVCS, aortic valve calcium score; AVI, aortic valve intervention; HG AS, high-gradient AS; HR, hazard ratio; LGAS, low-gradient AS.

Applying different AVCS cut-off values in the LGAS cohort, we found that in patients with AVCS values of above 1000 for women and 1800 for men and below 1400 for women and 2200 for men, AVI remained a significant predictor of better outcome (*Figure 4*).

## Discussion

In this single-centre retrospective propensity score–matched study, we investigated the outcomes of patients with severe AS and various aortic valve calcification loads undergoing AVI or medical treatment. The central findings of our study are that 1, AVCS was an independent predictor of mortality among patients with HG but not among those with LGAS; 2, AVI was a strong independent predictor of reduced mortality across the entire spectrum of patients with AS, including those with LGAS and a low AVCS, independent of the LVEF, with similar beneficial effect in the various groups.

There are two fundamental interpretations of our results. First, AVCS might not, in all instances, reliably reflect the true severity of AS and/or secondly, even patients with non-severe but symptomatic LGAS might benefit from AVI, especially when TAVI is performed.

### AVCS in patients with LGAS

LGAS poses a diagnostic challenge. For patients with LGAS and reduced LVEF, traditionally, dobutamine stress echocardiography (DSE) is recommended for further evaluation of AS severity. However, in addition to the limited availability of this modality, it encounters important technical limitations. It is dependent on a contractile reserve, and it involves several parameters that need to be assessed, inherently increasing the risk of measurement errors. Recent studies have questioned the utility of DSE in classical LFLG AS, reporting poor sensitivity for detecting severe AS and poor correlation with clinical outcomes.^11,12^ Moreover, the value of DSE among patients with PLFLG and NFLG AS is even less established.

Accordingly, CT-derived AVCS, which is a simple, highly reproducible, flow-independent quantitative method, has emerged as an important contributor to clinical decision-making with regard to the indication and timing for valve replacement.^4,13^ The diagnostic thresholds that identify severe AS were established in patients with HGAS. These thresholds are well reproduced in various studies providing similar diagnostic yield with a sensitivity of around 90%.^5,14^ This means that approximately one in every ten patients with clearly severe AS on echocardiography yet has a low AVCS. This discordance might be attributable to a greater component of fibrosis rather than calcification in the valve degeneration process in these patients^15,16^ because of various underlying pathologies. Rheumatic disease appears to damage the aortic valve without massive calcium accumulation.^17^ Data regarding the effect of bicuspid morphology on AVC load are controversial, suggesting that the relationship between aortic valve morphology and the degree of calcification is complex and is influenced by many factors, including age and sex.^7,17–19^ Although in younger women with bicuspid valves, the AVCS often underestimates AS severity, in older bicuspid patients, the degree of AVC correlates well with the haemodynamic measures of AS severity; furthermore, older men with bicuspid valves tend to have even higher calcification than their tricuspid peers. In the present cohort, patients with bicuspid aortic valve tended to have a higher AVCS than those with tricuspid valves, although the difference was not statistically significant, and none of the HG patients with a low AVCS had a bicuspid aortic valve.

Although the currently applied AVCS thresholds were established in HGAS, the real clinical utility of AVCS is, however, among patients with echocardiographically ambiguous LGAS. In our cohort, the rate of prevalence of a low AVCS was much higher among patients with LGAS (40%) than among those with HGAS (10%). This is in accordance with previous studies reporting that up to 50% of patients with LGAS may have below cut-off AVCS values.^5,14^

A high AVCS is a powerful tool to corroborate the diagnosis of severe AS in uncertain cases. On the other hand, whether a low AVCS represents pseudo-severe stenosis in all patients with LGAS, or patients with a low AVCS and LGAS constitute a heterogeneous cohort, including some cases with less calcific but severe AS, remains unclear.

The coexistence of CA with AS in elderly patients is not uncommon, and up to 30% of the LGAS population, and particularly PLFLG AS patients, may also have CA.^20^ It has been suggested that CA is associated with a lower AV calcification load;^8,21^ thus, it can be hypothesized that patients with CA and AS might constitute an important subgroup with a low AVCS despite severe AS, encountering particularly poor outcomes without the treatment of both conditions.

Whatever the underlying cause is, it appears that in patients with LGAS, AVCS might more frequently underestimate the severity of AS than in those with HGAS, which may delay appropriate management and impart a worse prognosis. The latter concept is supported by our result that among patients with LGAS, the outcome of patients with a low AVCS was not better than that of patients with a high AVCS; the prognostic value of AVCS was limited to patients with HGAS.

Several previous publications demonstrated that, across a spectrum of AS severity, a higher AVCS was associated with a higher risk of AVR or death.^6,22,23^ However, data on the predictive value of AVCS, specifically among patients with severe LGAS, are scarce. Aksoy and colleagues found that among severe LFLG AS patients with LVEF < 25%, an AVCS above the group median (2027AU, independent of sex) was associated with a higher risk of death.^24^ However, patients with PLFLG and NFLG AS were not included in that study, and the applied AVCS cut-off value was much higher than the guideline-recommended values for female patients. In another study, severe aortic valve calcification (defined as a calcium score ≥ 1200 in women and ≥ 2000 in men) was predictive of AVR or death in patients with PLFLG but not classical LFLG AS.^6^ A third study including patients with preserved LVEF and at least moderate AS found that although a higher AVCS (best cut-off values defined as ≥1577 AU for women and ≥2238 AU for men) did carry a significant prognostic value in patients with discordant grading AS, its prognostic impact was much weaker than in concordant grading moderate to severe AS.^5^ The more robust prognostic value of AVCS in HG than in LGAS suggests that in the latter subset of patients, AVCS might less reliably reflect AS severity. This concept is confirmed by a very recent study that evaluated the diagnostic accuracy of AVCS specifically in LGAS (92% classical LFLG), using DSE as a gold standard. In this report, it was found that AVCS was unable to distinguish true severe and pseudo-severe/moderate AS, exhibiting sensitivity and specificity values of only 44.3% and 56.5% respectively, along with positive and negative predictive values for severe AS of 50.0% and 50.8%, respectively. DSE-determined AS severity but not the AVCS was associated with clinical outcome regardless of AVR during follow-up.^25^

Our data demonstrate that irrespective of the true severity of AS, symptomatic patients with severe LGAS importantly benefit from AVI even if their aortic valve calcium load is relatively low.

### The impact of AVI among patients with a low AVCS

The decision about valve replacement in AS is based on the balance between the estimated risk of the procedure vs. the natural history of the disease. Although often asymptomatic, even moderate AS has been associated with increased mortality,^7,26,27^ especially in the context of impaired systolic LV function.^28–30^ Because of the inherently increased operative risk of these patients, the decision about AVR used to be a very delicate question. However, the appearance of TAVI transformed AVR into a much less invasive procedure, suitable even for higher-risk and more fragile patients including those with heart failure. Recent data suggest that in patients with impaired LV function, AVR is associated with survival benefit even in the case of moderate or pseudo-severe AS,^5,31,32^ especially when TAVI is performed. Jean and colleagues found significantly increased mortality in patients with moderate AS and reduced LVEF when compared with patients with heart failure alone, which was largely alleviated by AVR.^32^ An analysis of the TOPAS registry provided similar results, reporting a significant survival benefit with AVR when compared with medical treatment in patients with pseudo-severe AS.^33^ Finally, a recent large retrospective study by Ludwig and colleagues, including over 700 individuals with AS and reduced EF, confirmed that even among patients with pseudo-severe AS by CT, TAVI represented a major predictor of superior survival.^31^

If low aortic valve calcification load is taken as an indicator of pseudo-severe stenosis, our results are in accordance with the aforementioned findings. Whether patients with reduced LVEF and moderate AS actually benefit from AVR is currently being investigated in a large ongoing randomized-controlled trial (TAVR UNLOAD study).^34^ Until the results of this trial are revealed, the benefit of AVR in patients with impaired LV function and moderate AS remains unclear, and there is an even more substantial gap in evidence regarding whether afterload resolution by AVI could be beneficial to patients with preserved EF and pseudo-severe LGAS. In our study, less than half of the LGAS cohort had reduced LV function; nonetheless, AVI was associated with significantly reduced mortality even when the AVCS was below the guideline-recommended threshold. In an attempt to identify a different cut-off value below which AVI does not any more carry a survival benefit, we found that even in the case of an AVCS of 1000 AU in women and 1800 in men, AVI was still linked with a better survival when compared with conservative treatment. Whether the lack of survival benefit form AVI at thresholds more towards the margins of the AVCS spectrum is simply attributed to a lack of statistical power at lower case numbers or reflects a real biological effect, i.e. that AS with such a low calcification load is a benign condition, and AVVR is not necessary in these patients, remains to be clarified in larger-scale studies. It must be noted that, at least in the case of higher AVCS cut-offs, the latter explanation appears rather unlikely.

The clinical and imaging criteria that define the indication for valve replacement were established before TAVI was generally available. The diminished risk associated with this procedure might allow for lowering our thresholds to consider patients for AVR. The clear prognostic benefit of AVI among patients with a low AVCS indicate that these patients should not be denied invasive treatment based on a lower aortic valve calcium load. Further prospective studies are needed to determine whether the currently recommended AVCS cut-off values should be lowered to allow earlier intervention for symptomatic patients with LGAS.

### Limitations

The most important limitation of our study is the sample size, especially the fact that the number of LGAS patients was limited, which prevented reliable separate analyses of the various LGAS (LFLG, PLFLG, and NFLG) subsets. Nonetheless, the robustness of our results justifies this cohort size. Further larger-scale, prospective studies are warranted that allow a dissection of the observed results according to various LGAS subtypes. All analyses were performed in a retrospective manner, and therefore, no causal conclusions can be drawn from their results. Although all patients were recommended intervention, some selection bias with regard to why certain patients refused intervention cannot be completely ruled out. However, a comparison of the patients undergoing different treatments demonstrated essentially similar characteristics. DSE results were not available for all patients with classical LFLG AS; however, this approach is similar to that applied in previous studies^31^ and also represents a real-world practice. This was a single-centre study with all inherent limitations of such a study; on the other hand, it also ensured very uniform data (all echocardiography examinations performed by one of four echocardiographers, AVCS measured by a single investigator, clinical decisions made by the same heart team, and all interventions performed by the same pair of operators).

## Conclusion

The prevalence of a low AVCS is much higher among patients with LGAS than among those with HGAS. Among patients with symptomatic severe LGAS, a low AVCS does not entail a better prognosis compared with a high AVCS. AVI is equally beneficial in both the low and the high AVCS subgroups of LGAS, independent of the LV function, similarly to HGAS.

## Supplementary data

Supplementary data are available at *European Heart Journal - Cardiovascular Imaging* online.

## Supplementary Material

jeae276_Supplementary_Data

## Data Availability

I confirm that I will provide the underlying data for the purposes of peer review if these are requested.
