# Supplementary material for: Prognostic and therapeutic implications of a low aortic valve calcium score in patients with low-gradient aortic stenosis
Source: Eur Heart J Cardiovasc Imaging. 2024 Oct 29;26(2):287–98. doi: 10.1093/ehjci/jeae276 (PMC11781834; doi:10.1093/ehjci/jeae276)
Supplement: jeae276_Supplementary_Data [file jeae276_supplementary_data.zip › FINAL Revised Supplement MUR.docx]

**Table S1. Baseline demographic, clinical and imaging data of the patient cohort, stratified by the aortic mean transvalvular gradient, and by the LVEF, or the type of AS (LV EF and SVi) in case of HG and LG AS, repectively.**

|  | **HGAS**  **(n=243)** | |  | **LGAS**  **(n=84)** | | |  |
| --- | --- | --- | --- | --- | --- | --- | --- |
|  | **EF ≥ 50%**  **(n=93)** | **EF < 50%**  **(n=50)** | **p-value** | **LFLG**  **(n=40)** | **PLFLG**  **(n=11)** | **NFLG**  **(n=33)** | **p-value** |
| **Demographic data and co-morbidities** |  |  |  |  |  |  |  |
| **Age (years)** | 79.0 (± 7.0) | 78.5 (± 8.1) | 0.670 | 75.0 (± 8.4) | 77.5 (± 7.0) | 79.9 (± 5.5) | **0.004** |
| **Female (n, %)** | 108 (56.0) | 25 (50.0) | 0.453 | 9 (22.5) | 7 (63.6) | 17 (51.5) | **0.009** |
| **Hypertension (n, %)** | 167 (88.4) | 44 (88.0) | 0.944 | 37 (92.5) | 9 (81.8) | 31 (93.9) | 0.862 |
| **Diabetes mellitus (n, %)** | 75 (39.7) | 21 (42.0) | 0.767 | 25 (62.5) | 6 (54.5) | 15 (45.5) | 0.149 |
| **Atrial fibrillation (n, %)** | 73 (38.6) | 18 (36.0) | 0.735 | 20 (50.0) | 9 (81.8) | 12 (36.4) | 0.287 |
| **CAD (n, %)** | 63 (34.6) | 23 (47.9) | 0.091 | 22 (55.0) | 4 (36.4) | 9 (28.1) | **0.021** |
| **eGFR (mL/min/1.73m^2^)** | 61.6 (± 19.3) | 55.8 (± 21.2) | 0.064 | 55.5 (± 21.8) | 52.7 (± 26.2) | 58.1 (± 20.6) | 0.633 |
| **Echocardiography data** |  |  |  |  |  |  |  |
| **AMG (mmHg)** | 55.0 (± 14.9) | 50.8 (± 10.0) | 0.059 | 29.3 (± 7.4) | 29.3 (± 6.4) | 33.2 (± 6.0) | **0.019** |
| **AVA (cm^2^)** | 0.70 (± 0.19) | 0.60 (± 0.17) | **0.002** | 0.75 (± 0.20) | 0.77 (± 0.24) | 0.85 (± 0.14) | **0.029** |
| **LV EF (%)** | 60.1 (± 6.6) | 39.1 (± 8.4) | **<0.001** | 33.0 (± 6.8) | 57.8 (± 5.8) | 49.2 (± 13.7) | **<0.001** |
| **CT data** |  |  |  |  |  |  |  |
| **AVCS men (AU)** | 3764  (2516-4891) | 4041  (2597-5589) | 0.523 | 2030  (1662-3032) | 1772 (1384-2780) | 2860  (1689-3121) | 0.514 |
| **AVCS women (AU)** | 2240  (1665-3415) | 2081  (1715-3330) | 0.915 | 1128  (977-1339) | 1505  (1218-2596) | 1483  (1222-2592) | 0.218 |
| **Low AVCS (n, %)** | 22 (11.4) | 3 (6.0) | 0.265 | 20 (50.0) | 4 (36.4) | 10 (30.3) | 0.088 |
| **Survival after CT (days)** | 1838  (862-2167) | 1430  (518-2065) | 0.205 | 1789  (374-1976) | 644  (242-2057) | 1836  (848-2049) | 0.261 |

Continuous data are presented as mean ± standard deviation or median and interquartile ranges, while categorical variables are shown as numbers and percentages. Variables were compared between groups using Welch-corrected F-tests, except for survival after CT where Kruskal-Wallis test was used. In the latter case, raw survival times are presented; comparison of survival under medical treatment gave essentially identical results.

**HGAS:** AMG ≥40 mmHg and AVA ≤1 cm^2^ or AVAi ≤0.6 cm^2^/m^2^; **LGAS:** AMG <40 mmHg, AVA ≤1 cm^2^ or AVAi ≤0.6 cm^2^/m^2^.

AS, aortic stenosis; HGAS, high-gradient AS; LGAS, low-gradient AS; AVCS, aortic valve calcium score; CAD, coronary artery disease; eGFR, estimated glomerular filtration rate; AMG, aortic mean gradient; AVA, aortic valve area; LV EF, left ventricular ejection fraction; SVi, left ventricular stroke volume indexed to body surface area; CT, computed tomography; AU, Agatson unit.

**Table S2 Logistic regression coefficients for low AVCS.**

| **Predictor** | **OR** | **CI** | **p-value** |
| --- | --- | --- | --- |
| Age | 1.032 | 0.979-1.089 | 0.244 |
| Female | 0.631 | 0.294-1.353 | 0.236 |
| LV EF | 1.038 | 1.006-1.070 | **0.020** |
| Hypertension | 0.869 | 0.246-3.070 | 0.828 |
| Diabetes mellitus | 1.016 | 0.499-2.069 | 0.966 |
| Atrial fibrillation | 0.740 | 0.361-1.515 | 0.410 |
| CAD | 2.881 | 1.375-6.034 | **0.005** |
| eGFR | 0.982 | 0.965-1.001 | 0.057 |
| AVA | 1.355 | 0.178-10.320 | 0.769 |
| AMG | 0.888 | 0.852-0.927 | **<0.001** |

AVCS, aortic valve calcium score; OR, odds ratio; CI, confidence interval; LVEF, left ventricular ejection fraction; CAD, coronary artery disease; eGFR, estimated glomerular filtration rate; AVA, aortic valve area; AMG, aortic mean gradient.

**Table S3. Predicted median survival of patients with low and high AVCS, applying various cut-off values**

**A, Entire cohort**

| AVCS cut-off (Female/Male) | Low-gradient AS | | | High-gradient AS | | |
| --- | --- | --- | --- | --- | --- | --- |
|  | Low AVCS | High AVCS | p-value | Low AVCS | High AVCS | p-value |
| 800/1600 | 1732 | 1833 | 0.924 | 2216 | 1761 | 0.496 |
| 1200/2000 | 1273 | 2051 | 0.279 | 2693 | 1659 | **0.044** |
| 1600/3000 | 1712 | 2051 | 0.490 | 2036 | 1670 | 0.401 |
| 2000/2800 | 1662 | 2051 | 0.434 | 1912 | 1662 | 0.469 |

**B, Interventionally treated patients**

| AVCS cut-off (Female/Male) | Low-gradient AS | | | High-gradient AS | | |
| --- | --- | --- | --- | --- | --- | --- |
|  | Low AVCS | High AVCS | p-value | Low AVCS | High AVCS | p-value |
| 800/1600 | 1869 | 2051 | 0.819 | 2486 | 2023 | 0.324 |
| 1200/2000 | 1833 | 2059 | 0.526 | 2693 | 1907 | **0.034** |
| 1600/3000 | 1881 | 2059 | 0.653 | 2109 | 2023 | 0.594 |
| 2000/2800 | 1869 | 2059 | 0.598 | 2050 | 2029 | 0.835 |

AS, aortic stenosis; AVCS, aortic valve calcium score. The tested AVCS thresholds for men were always set 800 AU higher than for women.

**Table S4. Predictive value of aortic valve calcium score for survival under medical treatment (i.e., patients censored at aortic valve intervention) among patients with high-gradient aortic stenosis and low-gradient aortic stenosis.**

**A, High-gradient aortic stenosis**

|  | **Univariate** | | **Multivariate** | |
| --- | --- | --- | --- | --- |
| **Variables** | **HR (CI)** | **p-value** | **HR (CI)** | **p-value** |
| **Age (years)** | 0.969 (0.914-1.027) | 0.284 | 0.953 (0.886-1.025) | 0.197 |
| **Female sex** | 1.240 (0.530-2.903) | 0.620 | 3.103 (0.887-10.854) | 0.076 |
| **LV EF (%)** | 0.994 (0.957-1.032) | 0.761 | 0.988 (0.943-1.035) | 0.610 |
| **Hypertension** | 0.583 (0.165-2.055) | 0.401 | 0.496 (0.119-2.075) | 0.337 |
| **Diabetes mellitus** | 2.254 (0.862-5.893) | 0.097 | 2.362 (0.811-6.877) | 0.115 |
| **Atrial fibrillation** | 2.093 (0.814-5.378) | 0.125 | 1.621 (0.547-4.799) | 0.383 |
| **CAD** | 1.142 (0.461-2.830) | 0.774 | 1.892 (0.613-5.843) | 0.268 |
| **eGFR (mL/min/1.73m^2^)** | 0.995 (0.973-1.019) | 0.698 | 0.993 (0.965-1.022) | 0.642 |
| **AVCS [100 AU]** | 1.021 (1.008-1.034) | **0.001** | 1.035 (1.018-1.052) | **<0.001** |

**B, Low-gradient aortic stenosis**

|  | **Univariate** | | **Multivariate** | |
| --- | --- | --- | --- | --- |
| **Variables** | **HR (CI)** | **p-value** | **HR (CI)** | **p-value** |
| **Age (years)** | 1.014 (0.933-1.103) | 0.737 | 1.034 (0.902-1.185) | 0.633 |
| **Female sex** | 2.074 (0.552-7.795) | 0.280 | 1.293 (0.195-8.562) | 0.790 |
| **LV EF (%)** | 0.981 (0.929-1.037) | 0.498 | 0.976 (0.907-1.050) | 0.514 |
| **Hypertension** | 0.652 (0.075-5.639) | 0.698 | 1.775 (0.116-27.073) | 0.680 |
| **Diabetes mellitus** | 2.244 (0.542-9.293) | 0.265 | 1.809 (0.241-13.603) | 0.565 |
| **Atrial fibrillation** | 0.541 (0.134-2.176) | 0.387 | 0.627 (0.091-4.296) | 0.634 |
| **CAD** | 1.756 (0.418-7.371) | 0.442 | 0.959 (0.119-7.726) | 0.969 |
| **eGFR (mL/min/1,73m^2^)** | 0.961 (0.920-1.004) | 0.077 | 0.956 (0.908-1.007) | 0.087 |
| **AVCS [100 AU]** | 0.919 (0.825-1.023) | 0.124 | 0.892 (0.753-1.055) | 0.182 |

**HGAS:** AMG ≥40 mmHg and AVA ≤1 cm^2^ or AVAi ≤0.6 cm^2^/m^2^; **LGAS:** AMG <40 mmHg, AVA ≤1 cm^2^ or AVAi ≤0.6 cm^2^/m^2^.

AS, aortic stenosis; HGAS, high-gradient AS; AMG, mean transvalvular gradient; AVA, aortic valve area; AVAi, aortic valve area indexed to body surface area; LGAS, low-gradient AS; AU, Agatson unit; HR, hazard ratio; CI, confidence interval; LV EF, left ventricular ejection fraction; CAD, coronary artery disease; eGFR, estimated glomerular filtration rate; AVCS, aortic valve calcium score.

**The predictive value of low AVCS in patients with classical low flow low gradient, versus paradoxical low flow low gradient, versus normal flow low gradient AS**

Separate analysis of the predictive value of AVCS in the various LGAS subsets (LFLG, PLFLG, NFLG) revealed, that while the AVCS was not prognostic among patients with LFLG and NFLG AS, interestingly, it entailed a significant prognostic value among patients with PLFLG AS (**Figure S1**).

However, due to the low number of patients in the LGAS subgroups, these results should be interpreted with caution. Further, larger scale studies are warranted to clarify the prognostic importance of AVCS in various types of LGAS.

**Figure S1. The predictive value of AVCS (A to C) and the prognostic benefit of AVI (D to F) in various LGAS subsets. A and D**, classical LFLG AS; **B and E**, PLFLG AS; **C and F**, NFLG AS. Patients are stratified according to high or low AVCS.

AVCS, aortic valve calcium score; AVI, aortic valve intervention; LFLG AS, classical low flow low gradient AS; PLFLG, paradoxical low flow low gradient; NFLG, normal flow low gradient.

**Table S5. Baseline demographic, clinical and imaging data of the propensity score matched cohort.**

|  | **AVI**  **(n=183)** | **Medical therapy**  **(n=36)** | **p-value** |
| --- | --- | --- | --- |
| **Demographic data and co-morbidities** |  |  |  |
| **Age (years)** | 79.2 (± 6.7) | 79.4 (± 7.3) | 0.867 |
| **Female (n, %)** | 106 (57.9) | 20 (55.6) | 0.794 |
| **BMI (kg/m^2^)** | 27.7 (± 4.7) | 28.2 (± 6.1) | 0.628 |
| **Hypertension (n, %)** | 163 (89.1) | 32 (88.9) | 0.975 |
| **Diabetes mellitus (n, %)** | 80 (43.7) | 16 (44.4) | 0.936 |
| **Atrial fibrillation (n, %)** | 76 (41.5) | 14 (38.9) | 0.770 |
| **CAD (n, %)** | 71 (38.8) | 16 (44.4) | 0.529 |
| **eGFR (mL/min/1.73m^2^)** | 57.3 (± 20.5) | 55.9 (± 23.5) | 0.720 |
| **LV EF <50% (n, %)** | 59 (32.2) | 12 (33.3) | 0.899 |
| **Echocardiography data** |  |  |  |
| **AMG (mmHg)** | 50.8 (± 16.2) | 44.6 (± 15.8) | 0.035 |
| **AVA (cm^2^)** | 0.70 (± 0.18) | 0.70 (± 0.21) | 0.905 |
| **AVAi (cm^2^/m^2^)** | 0.37 (± 0.10) | 0.39 (± 0.13) | 0.461 |
| **LV EF (%)** | 53.0 (± 12.9) | 51.5 (± 13.9) | 0.538 |
| **CT data** |  |  |  |
| **AVCS (AU)** | 2487 (1748-3988) | 2496 (1684-3873) | 0.772 |
| **Low AVCS (n, %)** | 31 (16.9) | 8 (22.2) | 0.451 |
| **Survival after CT (days)** | 1844 (931-2172) | 285 (99-1060) | **<0.001** |

Continuous data are presented as mean ± standard deviation or median and interquartile ranges, while categorical variables are shown as numbers and percentages.

AVCS, aortic valve calcium score; BMI, body mass index; CAD, coronary artery disease; eGFR, estimated glomerular filtration rate; AMG, aortic mean gradient; AVA, aortic valve area; LV EF, left ventricular ejection fraction; CT, computed tomography; AU, Agatson unit.
