# Supplementary figures and images for: Prognostic and therapeutic implications of a low aortic valve calcium score in patients with low-gradient aortic stenosis
Source: Eur Heart J Cardiovasc Imaging. 2024 Oct 29;26(2):287–98. doi: 10.1093/ehjci/jeae276 (PMC11781834; doi:10.1093/ehjci/jeae276)

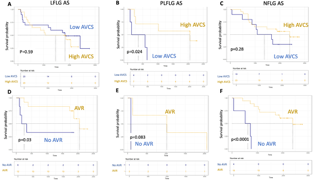

Supplement: jeae276_Supplementary_Data [file jeae276_supplementary_data.zip › Fig S1.tiff]
